# Supplementary material for: Nocturnal Transpiration May Be Associated with Foliar Nutrient Uptake
Source: Plants (Basel). 2023 Jan 24;12(3):531. doi: 10.3390/plants12030531 (PMC9919148; doi:10.3390/plants12030531)
Supplement: Supplementary file 1 [file plants-12-00531-s001.zip › plants-2042578-supplementary.pdf]

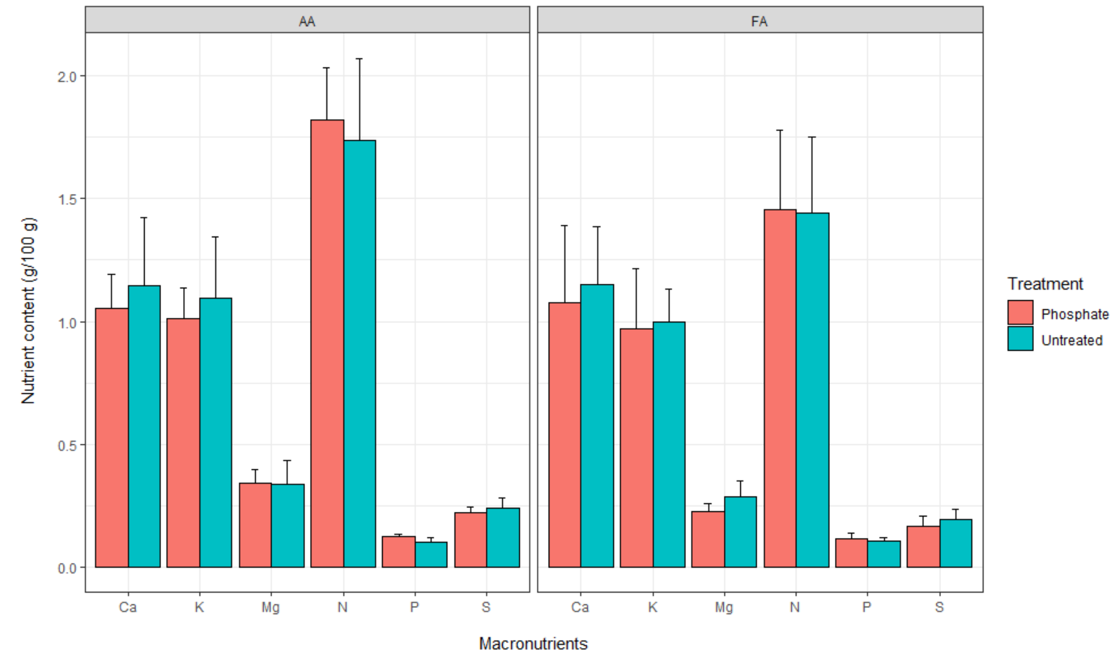

Figure S1: Concentration of macronutrients (g $100g^{-1}$ DW) of poplar leaves growing in AA (green bars in left figure) and FA (green bars in right figure). Red bars are foliar concentrations of plants treated with ammonium phosphate, respectively. The concentration of P data was compared using the Kruskal-Wallis test and the rest of the mineral concentration data were compared using ANOVA. For each analysis, the number of clones used was N = 8.

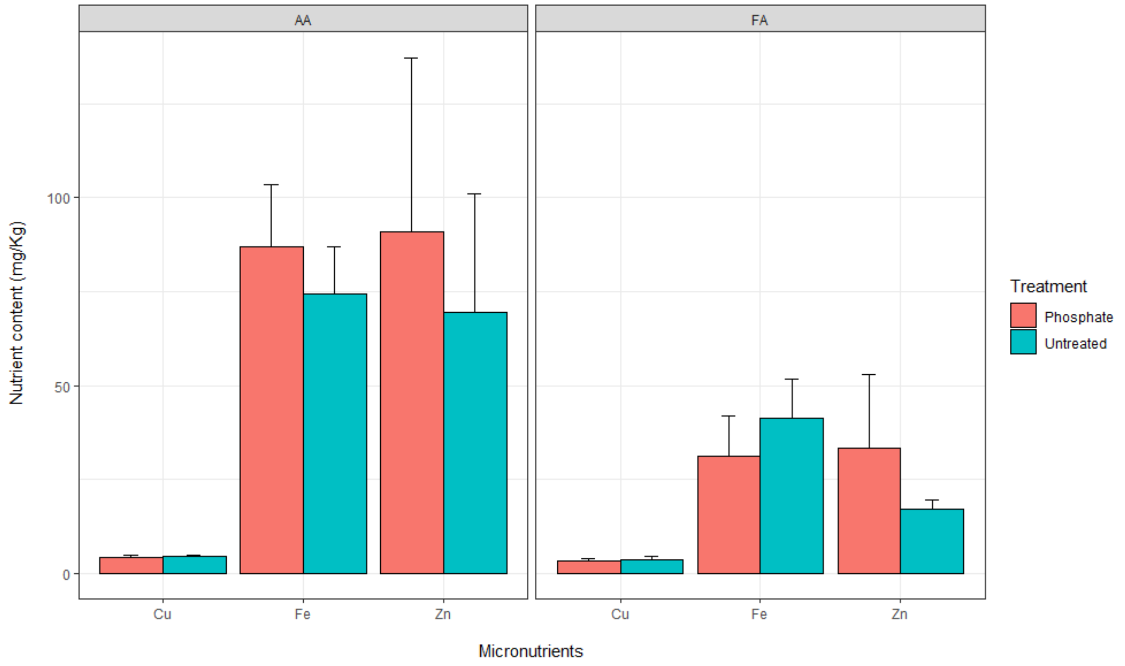

Figure S2: Concentration of micronutrients (mg Kg^{-1} DW) of poplar leaves growing in AA (green bars in left figure) and FA (green bars in right figure). Red bars are foliar concentrations of plants treated with ammonium phosphate, respectively. Zn concentration was compared using Yuen's test, and the rest of the mineral concentration data were compared using ANOVA. For each analysis, the number of clones used was N = 8.
